# A Retrospective before and after Assessment of Multidisciplinary Management for Postpartum Hemorrhage

**DOI:** 10.3390/jcm12237471

**Published:** 2023-12-02

**Authors:** Jarmila Anna Zdanowicz, Sophie Schneider, Carla Martignoni, Salima Lamari, Alexander Fuchs, Michael Daskalakis, Daniel Surbek

**Affiliations:** 1 Department of Obstetrics and Gynecology, Inselspital, Bern University Hospital, University of Bern, 3010 Bern, Switzerland; sophie.schneider@insel.ch (S.S.); daniel.surbek@insel.ch (D.S.); 2 Department of Anaesthesiology and Pain Medicine, Inselspital, Bern University Hospital, University of Bern, 3010 Bern, Switzerland; alexander.fuchs@insel.ch; 3 Department of Haematology and Central Haematology Laboratory, Inselspital, Bern University Hospital, University of Bern, 3010 Bern, Switzerland; michael.daskalakis@insel.ch

**Keywords:** hemostatic products, multidisciplinary management, patient blood management, postpartum hemorrhage, red blood cell transfusion

## Abstract

Postpartum hemorrhage (PPH) remains a major cause of maternal morbidity and mortality. While PPH treatment guidelines exist, data on their effect on reduction in red blood cell (RBC) transfusions and use of hemostatic products are scarce. Continuous evaluation of PPH management is important to assess potential pitfalls and incorporate new treatment options. We retrospectively compared PPH management and administration of RBC and hemostatic products before and after international guideline implementation. The primary endpoint was RBC administration for PPH. Secondary endpoints were hemoglobin trigger for RBC administration, administration of hemostatic products and surgical therapies. In total 235 patients had a PPH, 59 in 2011 and 176 in 2018. In 2018, fewer patients received RBC within 24 h (2018: 10% vs. 2011: 32%, *p* < 0.001) and 24 h after delivery (2018: 4.5% vs. 2011: 37%, *p* < 0.001). The number of RBC units transfused per case was significantly lower in 2018 (two vs. four units in 2011, *p* = 0.013). A significantly reduced transfusion of fresh frozen plasma and platelets was observed in 2018 (*p* < 0.001 and *p* = 0.002, respectively). In 2011, additional surgeries for PPH in both the acute and subacute phase were performed more frequently. Local implementation of multidisciplinary PPH guidelines is feasible and was associated with a significant reduction in transfused blood products.

## 1. Introduction

Postpartum hemorrhage (PPH) with a prevalence of up to 10% is one of the leading causes of maternal mortality worldwide, with a rate of approximately 25% [1,2,3]. In persistent severe PPH (defined as a blood loss of ≥1000 mL by the World Health Organization) complications such as severe hypovolemia, acute renal failure, hysterectomy, admission to an intensive care unit, postpartum sepsis, and maternal death are more common [4,5,6]. The transfusion of red blood cells (RBC) is a common treatment for acute hemorrhage-associated anemia, including PPH, to maintain adequate tissue oxygenation and perfusion. However, it has been shown that blood product transfusions also bear risks and can lead to adverse effects, including hemolytic transfusion reactions, transfusion-associated acute lung injury, transmission of infections, ischemic events, multiorgan failure, and transfusion-associated circulatory overload [7,8]. Consequently, these risks imply further economic drawbacks to hospitals and health care, such as additional surgical interventions, prolonged hospital stays, and further preventable medication costs [9,10].

Studies in medical specialties other than obstetrics have shown the advantage of restrictive RBC transfusions, including a lower hemoglobin transfusion trigger and fewer RBC units being administered [11,12,13]. This led to a more patient-centered use of blood products, so-called patient blood management (PBM). PBM aims to reduce blood loss, decrease unnecessary use of blood products, and to identify and treat anemia [14]. Several studies have demonstrated the beneficial outcomes of PBM implementation [15]. PBM practices impact blood loss and transfusion needs and reduce mortality, perioperative morbidity, hospital stay, and costs [16]. Although there are known risk factors for PPH, including placenta previa and previous PPH, up to two-thirds of women with a PPH have no known risk factors [5,6,17,18]. Iron deficiency anemia (IDA) occurs in almost 7% of all pregnant women and can be treated during pregnancy [19]. Women with IDA are at greater risk for peripartum RBC administration [20].

In 2012, the obstetric societies of Switzerland, Germany, and Austria developed a common PPH management guideline based on expert consensus, which was developed with specialists from obstetrics, anesthesia, and intensive care medicine to provide standardized management of PPH. This guideline includes pre-defined surgical and medical interventions and recommendations on patient blood management [21]. The obstetric societies conjointly amended this guideline in 2016, including updates on blood loss measurement, hemostatic treatment, and tranexamic acid administration [6]. Less is known about the effectiveness of this 2016 update in the reduction in blood product transfusion after implementation. PPH management should be reviewed on a regular basis to allow for improvement of existing guidelines and inclusion of new treatment options. This study aimed to analyze the impact of implementing PPH management guidelines on a departmental level, specifically its impact on blood product transfusions and use of hemostatic products.

## 2. Materials and Methods

### 2.1. Study Design

This retrospective before and after observational study was performed at the Department of Obstetrics at the University Hospital of Bern, a tertiary care center. The primary endpoint was defined as RBC administration for PPH. Secondary endpoints included the estimated hemoglobin trigger for RBC administration, administration of fresh frozen plasma (FFP) and platelet concentrates (PC), use of hemostatic products, and peripartal outcomes, including medical and surgical PPH treatments.

### 2.2. Patient Population and Inclusion Criteria

We included women who delivered at our hospital in 2011 and 2018 with a PPH. PPH was defined as blood loss of ≥500 mL after vaginal delivery and ≥1000 mL after Cesarean section (CS) [6]. We defined severe PPH as a blood loss of ≥1500 mL in the acute phase (within 24 h after delivery). To quantify blood loss, blood-soaked pads were weighed in cases of vaginal delivery. In cases of CS, blood loss was measured in the suction chamber. To ensure all patients with a PPH were included in our analysis, we screened our database using the following search criteria: postpartum hemorrhage, uterine atony, peripartal blood loss of at least 500 mL, placenta previa, placenta accrete/increta/percreta, Bakri Balloon (Cook medical, Bloomington, IN, USA), and red blood cell administration. We included a history of PPH in our search criteria to prevent missing potential patients with incomplete documentation. For our final detailed analysis, we only included patients with a PPH for the current delivery.

We specifically chose a year before PPH guideline development (2011) and effectively chose the first year after guideline update as well as with updated Swiss anemia guidelines in place (2018) [21]. Patients who delivered in an external hospital and were transferred postpartum to our tertiary care hospital for the treatment of PPH were also included. In addition, we identified patients for whom transfusion products were ordered at the Central Hematology Laboratory at our hospital during the immediate peripartum period or hospitalization after delivery. The amount of transfusion products received was retrieved from the patient records and used for analysis.

Demographic, pre-, peri-, and postpartum data were collected using the departmental database, which included patient history, laboratory records, medical records, histology records, imaging, and administrative data. We specifically examined pregnancy history and parameters related to PPH.

Regarding risk factors for PPH, we focused on those with an odds ratio of more than three according to the Association of Scientific Medical Societies in Germany (AWMF) guidelines [6]. This included previous PPH, preeclampsia (PE), HELLP syndrome (hemolysis, elevated liver enzymes, low platelet count), placental abruption, prolonged labor, placenta previa, increta, percreta, and accreta.

### 2.3. PPH Treatment

Regarding treatment for PPH, we examined acute (within 24 h postpartum) and subacute (more than 24 h postpartum but before hospital discharge after delivery) blood loss, transfused units of RBC, PC, FFP, medical treatment of PPH (including administration of uterotonics, tranexamic acid, fibrinogen, and clotting factors), and surgical procedures (including use of vaginal tamponade, Bakri Balloon insertion, artery embolization, sutures, and hysterectomy). Additionally, we examined hemoglobin levels during pregnancy, during the peripartal period, and before discharge after delivery.

In 2011 and 2018, we routinely administered 5IU oxytocin intravenously after spontaneous vaginal delivery (SVD) as well as instrumental vaginal delivery (IVD), and 100 mcg carbetocin intravenously after CS for uterine atony prophylaxis. In the case of PPH, we followed the standardized interdisciplinary PPH guidelines in 2018 [6]. Furthermore, treatment with tranexamic acid was administered earlier in 2018 for PPH, based on the results of the WOMAN trial [22,23].

### 2.4. Statistical Analysis

Statistical analyses were performed using SPSS version 25 (IBM Corp., Armonk, NY, USA). Continuous variables are presented as mean ± standard deviation, unless indicated as median with interquartile range. Categorical variables are presented as numbers and percentages. We calculated p-values using *t*-tests or Wilcoxon rank tests for continuous variables, as appropriate, and Fisher’s exact test or x^2^ test for categorical variables. To investigate whether PBM has an impact on the number of patients who received at least one RBC, we calculated odds ratios (OR) with 95% confidence intervals (CI) using logistic regression, first crude and then adjusted for the severity of PPH. We also calculated the association of known risk factors with the need for RBC, first crude and then adjusted for the year. Statistical significance was set at *p* < 0.05.

## 3. Results

Overall, 499 patients met our search criteria. 235 patients had a PPH (defined as a blood loss of ≥500 mL after vaginal delivery and ≥1000 mL after Cesarean section), 59 patients in 2011, and 176 patients during 2018, respectively. We had a total of 1398 deliveries at our clinic in 2011 and 2004 deliveries in 2018, corresponding to incidences of PPH of 4.2% (59/1398) in 2011 and 8.7% (176/2004) in 2018. No maternal deaths due to PPH were reported in 2011 or 2018.

### 3.1. Primary Endpoint

RBC administration to patients with PPH differed between 2011 and 2018 (Table 1). Fewer patients received RBC in 2018, both in the acute (10% in 2018 vs. 32% in 2011, *p* < 0.001) and subacute phases (4.5% in 2018 vs. 37% in 2011, *p* < 0.001). The number of RBC units transfused per case was significantly lower in 2018 (two units in 2018 vs. four units in 2011, *p* = 0.013.

Total blood loss, severe PPH, and RBC transfusion according to mode of delivery are presented in Table 2. The total blood loss and total amount of cases with severe PPH were significantly lower in 2018 compared to 2011 (1000 mL vs. 1500 mL, *p* < 0.001, and 19% vs. 39%, *p* = 0.005, respectively). Severe PPH and RBC administration during CS were significantly lower in 2018 compared to 2011 (41% vs. 71%, *p* = 0.024, and 24% vs. 67, *p* < 0.001, respectively).

We also performed multivariate analysis using RBC administration as the dependent variable. Overall, fewer patients received RBC in 2018 than in 2011 (odds ratio 0.12; *p* < 0.001). If adjusted for PPH severity (using a blood loss of ≥ 1500 mL as a cut-off), the effect of administering RBC more restrictively in 2018 was even slightly stronger, with an odds ratio of 0.09, *p* < 0.001, indicating that a reduction in RBC administration was most notably achieved in patients suffering from severe PPH. Adjusted for patient characteristics associated with a higher risk (including previous PPH, preeclampsia/HELLP, placental abruption, placenta previa, and bleeding/hematoma in pregnancy) for RBC transfusion, the risk of receiving RBC was only 1/8 in 2018 as compared to 2011.

### 3.2. Secondary Endpoints

#### 3.2.1. Hemoglobin Trigger for RBC Administration

In addition, the mean hemoglobin trigger level for RBC administration in the acute phase was slightly higher in 2018 with 75 g/L versus 72 g/L in 2011, but this was not significant (*p* = 0.55). On the other hand, the mean hemoglobin trigger level for RBC administration in the subacute phase was lower in 2018 with 67 g/L versus 70 g/L in 2011, however this difference was not significant.

#### 3.2.2. Administration of FFP, PC and Hemostatic Products

The use of hemostatic blood products for PPH is summarized in Table 3. In 2011 compared to 2018, platelets (14% vs. 2.3%, *p* = 0.002), FFP (34% in 2011 vs. 8.0% in 2018, *p* < 0.001), PC (14% vs. 2.3%, *p* = 0.002) and NovoSeven (Novo Nordisk, Bagsværd, Denmark) (5.1% vs. 0.0%, *p* = 0.015) were used significantly more often. Tranexamic acid was used more often in 2018 (70%) than in 2011 (58%), however, this difference was not significant.

#### 3.2.3. Surgical Treatment

Table 4 shows the surgical and interventional treatments used for PPH. In 2011, additional surgeries (both acute and subacute) were performed more frequently than in 2018; specifically, hysterectomies (5.1% vs. 0.0%, *p* = 0.015) and interventional embolizations (8.5% vs. 1.1%, *p* = 0.012) were performed significantly more often. In 2018, there was no recorded hysterectomy as a PPH treatment compared to the three cases in 2011. However, one case of planned hysterectomy without PPH was reported in 2018; hence, it was not included in our analysis. In cases of acute surgery (within 24 h after delivery), we included the most definitive surgery for each case in the final analysis when all other treatment options were exhausted. For example, in cases of dilatation and curettage (D&C) as well as tamponade insertion performed prior to peripartum hysterectomy, we only recorded hysterectomy as surgical treatment. For subacute surgery (>24 h after delivery but before hospital discharge after delivery), we only depicted severe cases that were treated in the operating room.

#### 3.2.4. Peripartal Outcomes

Table 5 summarizes the delivery characteristics and peripartum outcomes of patients. In 2011, uterine rupture (5.1% in 2011 vs. 0.0% in 2018, *p* = 0.015) and severe PPH (39% in 2011 vs. 19% in 2018, *p* = 0.005) occurred significantly more often.

The baseline characteristics are listed in Table 6. PPH patients in 2018 were significantly younger (32 + 4.8 years in 2018 vs. 33 + 4.6 in 2011, *p* = 0.018), had significantly more often singleton pregnancies (93% vs. 83%, *p* = 0.035), significantly less often a previous CS (8.5% vs. 24%, *p* = 0.005), and significantly more often a recorded IDA (30% vs. 3.4%, *p* < 0.001). Regarding PPH risk factors, as defined above, only the incidence of current PE/HELLP was significantly different between 2011 and 2018, with a higher incidence in 2011 (17% in 2011 vs. 7.4% in 2018, *p* = 0.043).

## 4. Discussion

Local implementation of the updated PPH algorithm starting in 2016 and its recommendation for PBM were associated with reduced RBC transfusion. Furthermore, a more restrictive transfusion practice after guideline implementation at our hospital was not associated with an increase in maternal morbidity or mortality. The incidence of PPH was twice as high in 2018 as in 2011, but the unplanned hysterectomy rate declined in 2018. This is most likely due to the increased awareness of, and consequently, better reporting of PPH in 2018, but also to fewer severe PPH cases in 2018. Furthermore, the risk of receiving RBC was only 1/8 in 2018 compared to 2011, indicating that the standardized interdisciplinary PPH guidelines implemented in 2016 that were in place in 2018 are likely to reduce the need for RBC administration. Surprisingly, hemoglobin transfusion triggers did not change.

In 2018, we administered RBC less frequently than in 2011, to minimize the potential adverse effects of RBC administration as reported [24]. While tranexamic acid is now routinely implemented as a first-line treatment of PPH as recommended in the WOMAN trial, this has not been done consistently in 2018 [22]. Furthermore, uterotonics were applied routinely after both vaginal delivery and Caesarean section in 2018, hence also decreasing the risk for uterine atony and PPH. In addition, significantly fewer RBC administrations in the subacute phase in 2018 could be due to better management in the acute phase, hence reducing the overall need. After PBM implementation, the use of hemostatic products in the management of PPH was lower, especially for FFP and thrombocytes. This might be explained by the slight tendency of increased tranexamic acid use in 2018 and, even though not recorded in this study, its early administration. More overall CS were recorded in 2011, but the median blood loss and the number of cases with severe PPH remains significantly higher after adjusting for mode of delivery. Furthermore, there were more multiple pregnancies and more previous CSs in 2011, which overall might also have contributed to more RBC administration in 2011.

The similar hemoglobin transfusion triggers in 2018 and 2011 might be due to several reasons. First, the decision to administer RBCs is based more on clinical symptoms than laboratory values, implying that a clinically unstable patient will receive RBC even if hemoglobin values are unavailable. Hence, hemoglobin levels are not routinely measured before RBC administration. Patients with an abnormally invasive placenta are often given RBC intraoperatively prophylactically in anticipation of excessive bleeding irrespective of hemoglobin; hence, the trigger to administer RBC might be higher. Furthermore, during the acute phase of PPH, hemoglobin levels were not always measured regularly by routine venous blood withdrawal but often by point-of-care testing with HemoCue (HemoCue AB, Ängelholm, Sweden), which was not always recorded in our electronic database. Consequently, the hemoglobin trigger level before RBC administration might not necessarily reflect the actual hemoglobin level and should be interpreted with caution in our study. Furthermore, similar hemoglobin transfusion triggers between 2011 and 2018 could be due to the increased awareness of PPH, resulting in faster treatment times. In addition, we routinely stored two units of RBC (universal application possible), fresh frozen plasma (FFP), and factor concentrate in the operating room.

The higher incidence of IDA and postpartum anemia recorded in 2018 was most likely due to better screening and documentation of iron deficiency [25]. Furthermore, PPH and its risk factors were better documented in 2018.

The need for effective PBM in obstetrics has already been recognized by anesthesiologists, and screening tools for critical care in obstetrics have been developed. Still, the focus remains mainly on the acute and subacute management of PPH [26,27,28]. A multidisciplinary approach involving disciplines involved in perioperative treatment is essential. While implementing a standardized multidisciplinary approach and managing severe PPH is beneficial, the emphasis seems to be on the use of tranexamic acid, RBC, FFP, fibrinogen, and other blood-related products [29,30,31]. While novel concepts exist, the focus remains mainly on PPH treatment [32]. Here, a multidisciplinary approach is crucial with PPH guidelines in place [28,33].

However, there has to be a shift towards better prevention, considering potential risk factors, but also optimizing care in the overall population, as most women with PPH have no known risk factors. Particularly, anemia in pregnancy is a significant risk factor for severe acute overall maternal morbidity and severe obstetric hemorrhage and transfusion, but also severe acute psychiatric disorders, independent of other risk factors [34]. This is an integral part of PBM guidelines in obstetrics and has been successfully implemented in Australia [25]. Furthermore, it has been shown that a single RBC unit protocol was able to avoid a second unit of packed red blood cells in more than 80% of women without a significant impact on morbidity [24]. In Switzerland and at our clinic specifically, we have been implementing improved anemia screening and treatment in pregnancy and have also seen a decrease in RBC administration despite an increase in PPH over the last years [35]. With standardized PPH management guidelines in most countries, there is a need for multidisciplinary PPH prevention and PBM implementation in obstetrics, which should be the focus of further PPH studies.

A strength of our study is that we are a tertiary care and national referral center with a clinical routine for treating complicated PPH cases. One limitation of our study is its retrospective nature, which makes it more likely to have incomplete data, including further classification of CS. Furthermore, our small number of cases, particularly severe PPH, is another limitation. The single-center design of the study might not be generalizable to other settings. Also, we realize that our definition of PPH applies to German-speaking countries. However, as PPH definitions vary across different societies and countries, we decided to apply the local definition to best reflect practices.

## 5. Conclusions

In summary, we were able to show that the implementation of multidisciplinary PPH treatment guidelines, including PBM recommendations, is feasible at the departmental level and was associated with significantly reduced RBC, FFP, and PC transfusions. Such audits to evaluate the effect of guideline implementation are crucial to investigate weaknesses and areas for improvement that might contribute to further multidisciplinary updates.

## Figures and Tables

**Table 1:** Characteristics regarding laboratory values and red blood cell administration in patients with PPH in 2011 and 2018.

	N	Patients with PPH	2011	2018	
		N = 235	N = 59	N = 176	*p*-Value
Hemoglobin level (g/l) before delivery	235	121 ± 13 (69, 172)	121 ± 13 (81, 158)	121 ± 13 (69, 172)	0.98
Platelet count (G/l) before delivery	212	198 ± 62 (22, 425)	193 ± 72 (62, 425)	200 ± 59 (22, 398)	0.33
Total patients with RBC (acute and/or subacute)	235	54 (23%)	32 (54%)	22 (13%)	<0.001 *
Amount of RBC units transfused	54	3.0 [2.0 to 5.0]	4.0 [2.0 to 6.0]	2.0 [2.0 to 4.0]	0.013 *
Acute (within 24 h after delivery) RBC administration	235	36 (15%)	19 (32%)	17 (10%)	<0.001 *
Hemoglobin trigger (g/l) before acute RBC administration	31	74 ± 15 (29, 110)	72 ± 19 (29, 97)	75 ± 12 (59, 110)	0.55
Hemoglobin level (g/l) after acute RBC administration	34	93 ± 13 (72, 135)	95 ± 15 (74, 135)	92 ± 12 (72, 111)	0.59
Subacute (after 24 h after delivery) RBC administration	235	30 (13%)	22 (37%)	8 (4.5%)	<0.001 *
Hemoglobin trigger (g/l) before subacute RBC administration	17	68 ± 10 (46, 86)	70 ± 8.4 (55, 82)	67 ± 12 (46, 86)	0.47
Hemoglobin level (g/l) after subacute RBC administration	17	89 ± 12 (67, 110)	93 ± 11 (78, 110)	85 ± 11 (67, 101)	0.17
Hemoglobin level (g/l) before discharge	235	96 ± 14 (68, 134)	97 ± 13 (73, 130)	96 ± 14 (68, 134)	0.48
Platelets count (G/l) before discharge	229	215 ± 74 (105, 563)	254 ± 107 (105, 563)	203 ± 56 (105, 373)	0.005 *
Postpartal anemia	235	164 (70%)	7 (12%)	157 (89%)	<0.001 *
Postpartal iron treatment (perorally)	235	184 (78%)	47 (80%)	137 (78%)	0.86
Postpartal iron treatment (outpatient intravenous administration)	235	68 (29%)	3 (5.1%)	65 (37%)	<0.001 *

PPH postpartum hemorrhage; RBC red blood cell. Data presented in mean ± standard deviation (min, max), n (%) or median [IQR]. * significant (*p* < 0.05).

**Table 2:** Blood loss, severity of PPH, and RBC transfusions according to the mode of delivery.

	Patients with PPH	2011	2018	
	N = 235	N = 59	N = 176	*p*-Value
Blood loss (mL) Spontaneous delivery	800 [600 to 1200]	1050 [700 to 1500]	800 [600 to 1000]	0.019 *
Instrumental delivery	800 [600 to 1100]	1100 [800 to 2000]	800 [600 to 1000]	0.11
Cesarean section	1500 [1000 to 2000]	2000 [1350 to 2750]	1200 [1000 to 1700]	0.002 *
Severe PPH ≥ 1500 mL	57/235 (24%)	23/59 (39%)	34/176 (19%)	0.005 *
Spontaneous delivery	17/138 (12%)	5/30 (17%)	12/108 (11%)	0.53
Instrumental delivery	4/27 (15%)	1/5 (20%)	3/22 (14%)	1.00
Cesarean section	36/70 (51%)	17/24 (71%)	19/46 (41%)	0.024 *
RBC administration (any)	54/235 (23%)	32/59 (54%)	22/176 (13%)	<0.001 *
Spontaneous delivery	23/138 (17%)	14/30 (47%)	9/108 (8%)	<0.001 *
Instrumental delivery	4/27 (15%)	2/5 (40%)	2/22 (9%)	0.14
Cesarean section	27/70 (39%)	16/24 (67%)	11/46 (24%)	<0.001 *

PPH postpartum hemorrhage; RBC red blood cell. Data presented in median [lq to uq] or n/N (%). * significant (*p* < 0.05).

**Table 3:** Combined (acute and subacute) hemostatic replacement therapies.

Hemostatic Therapy	Patients with PPH	2011	2018	
	N = 235	N = 59	N = 176	*p*-Value
Platelets (any)	12 (5.1%)	8 (14%)	4 (2.3%)	0.002 *
Platelet trigger (G/l) before PC administration	77 ± 40 (22, 157)	75 ± 22 (41, 106)	80 ± 63 (22, 157)	1.00
Platelet count (G/l) after PC administration	101 ± 37 (56, 172)	90 ± 15 (70, 108)	116 ± 53 (56, 172)	0.46
Fresh frozen plasma (any)	34 (14%)	20 (34%)	14 (8.0%)	<0.001 *
Tranexamic acid (any)	158 (67%)	34 (58%)	124 (70%)	0.08
Beriplex (any)	1 (0.43%)	0 (0.00%)	1 (0.57%)	1.00
Fibrinogen (any)	24 (10%)	10 (17%)	14 (8.0%)	0.08
Hemate (any)	1 (0.43%)	1 (1.7%)	0 (0.00%)	0.25
NovoSeven (any)	3 (1.3%)	3 (5.1%)	0 (0.00%)	0.015 *
Cell saver re-transfusion (any)	1 (0.43%)	1 (1.7%)	0 (0.00%)	0.25

PC platelet concentrate. Data presented in mean ± standard deviation (min, max) or n (%). * significant (*p* < 0.05).

**Table 4:** Surgical PPH treatment in 2011 and 2018.

	Patients with PPH	2011	2018	
	N = 235	N = 59	N = 176	*p*-Value
	n (%)	n (%)	n (%)	
Acute surgical PPH treatment (SVD/IVD)				0.46
Tamponade/Balloon (Bakri/Foley)	12 (4.2%)	7 (12%)	5 (2.8%)	
Embolization	2 (0.85%)	1 (1.7%)	1 (0.57%)	
Laparotomy/sutures	1 (0.42%)	1 (1.7%)	0 (0.00%)	
Acute additional surgical treatment (CS)				0.21
Tamponade/Balloon (Bakri/Foley)	9 (3.8%)	2 (3.4%)	7 (4.0%)	
Embolization	4 (1.7%)	4 (6.8%)	0 (0.00%)	
Sutures	7 (3.0%)	2 (3.4%)	5 (2.8%)	
Hysterectomy	3 (1.3%)	3 (5.1%)	0 (0.00%)	
Subacute D&C surgery for all MOD	6 (2.6%)	2 (3.4%)	4 (2.3%)	0.64
Subacute surgery for all MOD	6 (2.6%)	2 (3.4%)	4 (2.3%)	0.64
Tamponade/Balloon (Bakri/Foley)	2 (0.85%)	0 (0.00%)	2 (1.1%)	1.00
Laparotomy/Re-laparotomy/Sutures	2 (0.85%)	1 (1.7%)	1 (0.57%)	0.44
Embolization	1 (0.43%)	0 (0.00%)	1 (0.57%)	1.00

MOD mode of delivery; D&C dilation and curettage. Data presented in n (%).

**Table 5:** Delivery characteristics and peripartal outcomes with PPH in 2011 and 2018.

	Patients with PPH	2011	2018	
	N = 235	N = 59	N = 176	*p*-Value
Gestational age at delivery (weeks) N = 232	37 ± 5.4 (16, 42)	35 ± 6.8 (16, 42)	38 ± 4.6 (18, 42)	<0.001 *
Hospitalization before delivery	41 (17%)	14 (24%)	27 (15%)	0.17
Type of delivery				0.11
Spontaneous vaginal delivery	138 (59%)	30 (51%)	108 (62%)	
Instrumental vaginal delivery	27 (11%)	5 (8.5%)	22 (13%)	
Cesarean section	70 (30%)	24 (41%)	46 (26%)	
Peripartal complications				
Placental abruption	9 (3.8%)	3 (5.1%)	6 (3.4%)	0.69
Placental retention	81 (34%)	25 (42%)	56 (32%)	0.16
Placenta previa	25 (11%)	8 (14%)	17 (10%)	0.46
Uterine atony	100 (43%)	15 (25%)	85 (48%)	0.002 *
Uterine rupture	3 (1.3%)	3 (5.1%)	0 (0.00%)	0.015 *
Estimated total blood loss (mL)	1000 [700, 1500]	1500 [900, 2000]	1000 [700, 1200]	<0.001 *
Severe postpartum hemorrhage ≥ 1500 mL	57 (24%)	23 (39%)	34 (19%)	0.005 *
Neonatal data				
Birth weight (g) N = 232	3135 [2305, 3500]	2625 [1850, 3225]	3250 [2605, 3540]	<0.001 *
Birth weight ≥ 4000 g	14 (6.0%)	1 (1.7%)	13 (7.4%)	0.20
Intrauterine fetal death (>15 weeks of gestation)	7 (3.0%)	2 (3.4%)	5 (2.8%)	1.00
Neonatal intensive care unit (NICU)	58 (25%)	27 (46%)	31 (18%)	<0.001 *

Data presented in mean ± standard deviation (min, max), n (%) or median [lq to uq]. * significant (*p* < 0.05).

**Table 6:** Baseline characteristics of patients with PPH in 2011 and 2018.

	Patients with PPH	2011	2018	
	N = 235	N = 59	N = 176	*p*-Value
Maternal age (years) at birth	32 ± 4.8 (19, 44)	33 ± 4.6 (22, 44)	32 ± 4.8 (19, 43)	0.018 *
Parity				0.43
0	6 (2.6%)	3 (5.1%)	3 (1.7%)	
1–2	191 (81%)	45 (76%)	146 (83%)	
≥3	38 (16%)	11 (19%)	27 (15%)	
Singleton birth	213 (91%)	49 (83%)	164 (93%)	0.035 *
Previous Cesarean delivery	29 (12%)	14 (24%)	15 (8.5%)	0.005 *
Current preeclampsia/eclampsia/HELLP	23 (10%)	10 (17%)	13 (7.4%)	0.043 *
Prolonged labor	16 (6.8%)	5 (8.5%)	11 (6.3%)	0.56
Multiple pregnancy	22 (9.4%)	10 (17%)	12 (6.8%)	0.035 *
Medical condition				
Iron deficiency anemia	55 (23%)	2 (3.4%)	53 (30%)	<0.001 *
Bleeding / hematoma in pregnancy	24 (10%)	9 (15%)	15 (8.5%)	0.14
Congenital bleeding disposition	3 (1.3%)	1 (1.7%)	2 (1.1%)	1.00
Congenital thrombotic event disposition	6 (2.6%)	1 (1.7%)	5 (2.8%)	1.00
Cardiovascular disease	7 (3.0%)	1 (1.7%)	6 (3.4%)	0.68
Pre-existent hypertension	3 (1.3%)	2 (3.4%)	1 (0.57%)	0.16
Gestational diabetes	24 (10%)	6 (10%)	18 (10%)	1.00
PPH risk factors				
Previous postpartum hemorrhage	10 (4.3%)	5 (8.5%)	5 (2.8%)	0.13
Previous preeclampsia/eclampsia/HELLP	10 (4.3%)	6 (10%)	4 (2.3%)	0.018 *
Previous placental abruption	2 (0.85%)	1 (1.7%)	1 (0.57%)	0.44
Previous uterine atony	10 (4.3%)	5 (8.5%)	5 (2.8%)	0.13
Previous placental retention	11 (4.7%)	5 (8.5%)	6 (3.4%)	0.15
Previous placental previa	3 (1.3%)	0 (0.00%)	3 (1.7%)	0.57

Data presented in mean ± standard deviation (min, max) or n (%). * significant (*p* < 0.05).

## Data Availability

The data presented in this study are available on request from the corresponding author. The data are not publicly available due to privacy regulations.
